# *Magnolia* Extract (BL153) Protection of Heart from Lipid Accumulation Caused Cardiac Oxidative Damage, Inflammation, and Cell Death in High-Fat Diet Fed Mice

**DOI:** 10.1155/2014/205849

**Published:** 2014-02-16

**Authors:** Weixia Sun, Zhiguo Zhang, Qiang Chen, Xia Yin, Yaowen Fu, Yang Zheng, Lu Cai, Ki-Soo Kim, Ki Ho Kim, Yi Tan, Young Heui Kim

**Affiliations:** ^1^Departments of Nephrology and Cardiology, The First Hospital of Jilin University, Changchun 130021, China; ^2^Kosair Children's Hospital Research Institute, Department of Pediatrics, University of Louisville, Louisville, KY 40202, USA; ^3^Preventive Medicine School, Jilin University, Changchun 130021, China; ^4^The Chinese-American Research Institute for Diabetic Complications, The Wenzhou Medical University, Wenzhou 325035, China; ^5^Bioland Biotec Co., Ltd., Zhangjiang Modern Medical Device Park, Pudong, Shanghai 201201, China; ^6^Bioland R&D Center, 59 Songjeongni 2-gil, Byeongcheon, Dongnam, Cheonan, Chungnam 330-863, Republic of Korea

## Abstract

*Magnolia* as an herbal material obtained from *Magnolia officinalis* has been found to play an important role in anti-inflammation, antioxidative stress, and antiapoptosis. This study was designed to investigate the effect of *Magnolia* extract (BL153) on obesity-associated lipid accumulation, inflammation, oxidative stress, and apoptosis in the heart. C57BL/6 mice were fed a low- (10 kcal% fat) or high-fat (60 kcal% fat) diet for 24 weeks to induce obesity. These mice fed with high-fat diet (HFD) were given a gavage of vehicle, 2.5, 5, or 10 mg/kg body weight BL153 daily. The three doses of BL153 treatment slightly ameliorated insulin resistance without decrease of body weight gain induced by HFD feeding. BL153 at 10 mg/kg slightly attenuated a mild cardiac hypertrophy and dysfunction induced by HFD feeding. Both 5 mg/kg and 10 mg/kg of BL153 treatment significantly inhibited cardiac lipid accumulation measured by Oil Red O staining and improved cardiac inflammation and oxidative stress by downregulating ICAM-1, TNF-**α**, PAI-1, 3-NT, and 4-HNE. TUNEL staining showed that BL153 treatment also ameliorated apoptosis induced by mitochondrial caspase-3 independent cell death pathway. This study demonstrates that BL153 attenuates HFD-associated cardiac damage through prevention of HFD-induced cardiac lipid accumulation, inflammation, oxidative stress, and apoptosis.

## 1. Introduction 

In recent years, the number of the obese people increased significantly. Obesity typically results from overeating and lack of enough exercise, which is a growing global health problem [1]. Obesity is associated with many cardiovascular diseases (CVD). Hyperlipidemia, inflammation, oxidative stress, myocardial apoptosis, lipid metabolic disorder, and insulin resistance are all important pathological factors for the increased CVD in both diabetic and obese patients [2–4]. Currently, there are many therapeutic strategies for diabetes and its complications, such as glycemic management, diet control, and exercise [5]. However, no single approach is able to efficiently prevent obesity associated CVD.

Natural products with significant antidiabetes and antiobesity efficacy, but without toxic effects, are very attractive for the preventive and therapeutic purposes [6]. *Magnolia officinalis* is a natural product used for the empirical treatment of diabetes in Korea [7]. Honokiol (HON) and magnolol (MAG), 4-O-methylhonokiol and obovatol isolated from the stem bark of *Magnolia* plants, are considered as the major bioactive constituents [8]. Recently, the studies demonstrated that constituents of *Magnolia* such as HON and MAG have anti-inflammatory [9–11], -oxidative [12, 13], and -apoptotic effects [13, 14]. Moreover, another study showed that MAG reduced fasting blood glucose and plasma insulin levels in type 2 diabetic model without altering body weight [15] and increased glucose uptake in 3T3-L1 adipocytes [16]. However, the effects of *Magnolia officinalis* on heart of obesity induced by HFD still remain unclear.

In the present study, we investigated whether *Magnolia officinalis* extract prevents cardiac lipid accumulation, inflammation, oxidative stress, and apoptosis in the heart of obese mice induced by HFD.

## 2. Material and Methods

### 2.1. *Magnolia* Extract (BL153)

*Magnolia* extract (BL153) was prepared by Bioland Co., Ltd. (Chungnam, Korea) and dissolved in 0.5% ethanol as previously reported [17].

### 2.2. Animal Models

C57BL/6J male mice, 8 weeks of age, were purchased from the Jackson Laboratory (Bar Harbor, Maine) and housed in the University of Louisville Research Resources Center at 22°C with a 12 h light/dark cycle with free access to standard rodent chow and tap water. All experimental procedures for these animals were approved by the Institutional Animal Care and Use Committee of the University of Louisville, which is compliant with the Guide for the Care and Use of Laboratory Animals published by the US National Institutes of Health (NIH Publication no. 85-23, revised in 1996).

A Total of 25 mice were randomly assigned into 5 groups with 5 mice per group as follows: Ctrl (control) group, fed control diet of 10 kcal% from fat (D12450B, Research Diets Inc. 3.85 kcal/g) and given a gavage of the vehicle (0.5% ethanol); HFD group, fed the high-fat diet (HFD) of 60 kcal% from fat (D12492, Research Diets Inc. 5.24 kcal/g) and given a gavage of the vehicle; HFD + 2.5 mg/kg group, fed HFD and given a gavage of 2.5 mg/kg body weight of BL153; HFD + 5 mg/kg group, fed HFD and given a gavage of 5 mg/kg body weight of BL153; HFD + 10 mg/kg group, fed HFD and given a gavage of 10 mg/kg body weight of BL153. All mice were fed the corresponding diet and treated with BL153 or vehicle as described above simultaneously for 24 weeks. Energy intake and body weight were monitored daily or weekly, respectively. After insulin tolerance test and blood pressure and echocardiography measurements, mice were sacrificed. The hearts were isolated and weighted, and blood plasma was collected. Average energy intake was calculated following the diet formula (D12450B or D12492, Research Diets Inc.): per mice/day (kcal) = (food (g) intake/cage/day) × 3.85 (control diet) or 5.24 (HFD)/(mice/per cage).

### 2.3. Intraperitoneal Insulin Tolerance Test (IPITT)

IPITT was conducted after 24 weeks of high-fat diet feeding. For IPITT [18], mice (*n* = 5 per group) were fasted overnight (14 h), weighed, and then injected with human insulin (Humulin R; Eli Lilly, Indianapolis, IN) intraperitoneally at a dose of 1 unit/kg body weight. Blood glucose levels at 0, 15, 30, 60, and 120 min after insulin injection were measured using a FreeStyle Lite glucometer (Abbott Diabetes Care, Alameda, CA). Area under the curve (AUC) was calculated by the trapezoid rule for the insulin tolerance curve using Origin 7.5 software (OriginLab Corporation, Northampton, MA).

### 2.4. Noninvasive Blood Pressure (BP)

BP was measured by tail-cuff plethysmography using a CODA6 noninvasive BP monitoring system (Kent Scientific, Torrington, CT) as previously reported [19]. Mice (*n* = 5 per group) were restrained in a plastic tube restrainer. Occlusion and volume-pressure recording (VPR) cuffs were placed over the tail, and the mice were allowed to adapt to the restrainer for 5 min prior to starting BP measurement. After a 5 min adaptation period, BP was measured for 10 acclimation cycles followed by 20 measurement cycles. Mice were warmed by heating pads during the acclimation cycles to ensure sufficient blood flow to the tail. The animals were monitored closely throughout the measurement protocol and removed from restraint as soon as possible upon completing the measurement protocol. After three days of training for the BP measurement, formal measurements were performed and systolic, mean, and diastolic BP and heart rate data were collected.

### 2.5. Echocardiography

Transthoracic echocardiography (Echo) was detected for Avertin anesthetized mice by high-resolution imaging system (Vevo 770, VisualSonics, Canada) equipped with a high-frequent ultrasound probe (RMV-707B) as described in previous study [20]. The chests of the mice (*n* = 5 per group) were treated with a chemical hair remover to reduce ultrasound attenuation. Parasternal long-axis and short-axis views were acquired. Left ventricular (LV) dimensions and wall thicknesses were determined from parasternal short axis M-mode images. The anesthetized heart rate was collected. Meanwhile, ejection fraction (EF), fractional shortening (FS), and LV mass were calculated by Vevo770 software. The final data represent averaged values of 10 cardiac cycles.

### 2.6. Oil Red O Staining for Lipid Accumulation in Heart

Cryosections (10 *μ*m) from OCT-embedded heart tissue samples were fixed in 10% buffered formalin for 5 min and slides were washed in water. Then the slides were immersed in 60% isopropanol for 30 s and incubated in Oil Red O solution for 1 h at room temperature. The slides were washed with 60% isopropanol twice and then counterstained with hematoxylin (DAKO, Carpinteria, CA, USA) for 30 s. Excess hematoxylin was washed in water. A Nikon microscope (Nikon, Melville, NY) was used to capture the Oil Red O-stained tissue sections.

### 2.7. Plasma Triglyceride Quantification

Concentration of triglyceride in the plasma was measured according to the manufacturers' instruction provided in the triglyceride colorimetric assay kit (Cayman Chemical, CA).

### 2.8. Terminal Deoxynucleotidyl Transferase-Mediated dUTP Nick End Labeling (TUNEL) Assay

Apoptotic nuclei in the heart were examined by TUNEL staining using ApopTag In Situ kit (Chemicon, Temecula, CA). Heart tissue was fixed in buffered neutral 10% formalin, dehydrated in graded alcohol series, embedded in paraffin, and sectioned at 4 *μ*m. Deparaffinized and hydrated slides were used for TUNEL staining according to the manufacturer's instructions. TUNEL positive nuclei cells were counted under high power field (HPF, 20x) in five random fields for each of three slides from one mouse with at least five mice (as indicated) in each group and presented as TUNEL positive nuclei per HPF as described in previous study [21].

### 2.9. Western Blot Assay

Briefly, heart tissues were homogenized with RIPA lysis buffer (Santa Cruz Biotechnology, CA). Total proteins were extracted and separated on SDS-PAGE gels, and then proteins were transferred to a nitrocellulose membrane (Bio-Rad, Hercules, CA). The membrane was blocked with 5% nonfat dried milk for 1 h and then incubated overnight at 4°C with the following primary antibodies: intercellular adhesion molecule 1 (ICAM-1, Santa Cruz Biotechnology, CA), tumor necrosis factor alpha (TNF-*α*, Abcam, Cambridge, MA), plasminogen activator inhibitor-1 (PAI-1, BD Bioscience, Sparks, MD), 3-nitrotyrosine (3-NT, Millipore, Billerica, MA), 4-hydroxy-2-nonenal (4-HNE, Alpha Diagnostic International, San Antonio, TX), B-cell lymphoma 2 (BCL-2) and BCL2-associated X protein (BAX) (Cell Signaling, MA), cleaved-caspase-8 (Cell Signaling, MA), apoptosis inducing factor (AIF) (Cell Signaling, MA), caspase-12 (Exalpha Biologicals, MA), C/EBP homologous protein (CHOP; Santa Cruz Biotechnology, CA) GAPDH (Santa Cruz Biotechnology, CA), and cleaved-caspase-3 (Cell Signaling, MA). After three washes with Tris-buffered saline (pH 7.2) containing 0.1% Tween 20 (TBST), membranes were incubated with appropriate secondary antibodies for 1 h at room temperature. Antigen-antibody complexes were then visualized using an enhanced chemiluminescence kit (Thermo Scientific, Rockford, IL) [22].

### 2.10. Quantitative Analysis of Lipid Peroxides

The lipid peroxide concentration was measured by detecting thiobarbituric acid (TBA) reactivity reflected by the amount of malondialdehyde (MDA) formed during acid hydrolysis of the lipid peroxide compound. The reaction mixtures included 20 *μ*L 8.1% sodium dodecyl sulfate, 50 *μ*L protein sample, 210 *μ*L 0.571% TBA, and 150 *μ*L 20% acetic acid solution (pH 3.5). Each sample was duplicated. The reaction mixtures were incubated at 90°C for 1 h. After cooling down on ice, 100 *μ*L distilled water was added and centrifuged at 4000 rpm for 15 min. Then the absorbance of each sample at 540 nm was measured by using 150 *μ*L supernatant at a microplate reader.

### 2.11. Statistical Analysis

Data were collected from 5 mice per group and presented as means ± SD as indicated. We used Image Pro Plus 6.0 software (Media Cybernetics, Bethesda, MA) to identify the positive staining and Image Quant 5.2 software (GE Healthcare Bio-Sciences AB) to analyze Western blot. Comparisons were performed by one-way ANOVA for the different groups, followed by post hoc pairwise repetitive comparisons using Tukey's test with Origin 8.0 Lab data analysis and graphing software (Origin Lab Corporation, Northampton, MA). Statistical significance was considered as *P* < 0.05.

## 3. Results

### 3.1. The Effects of BL153 on Body Weight and Energy Intake in HFD Feeding Mice

**Figure 1:**
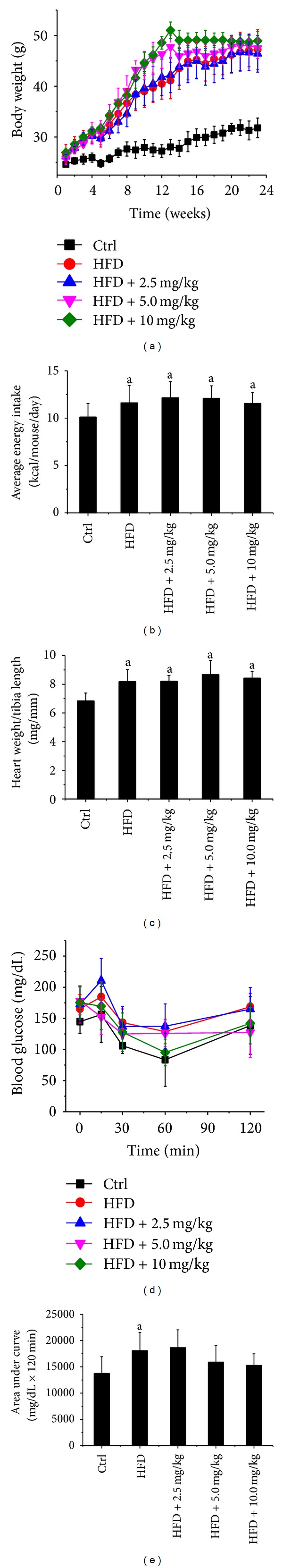
The effects of BL153 on body weight, energy intake, heart weight, and lung weight. Male C57BL/6J mice at 8 weeks of age were fed either a LFD (10% kcal as fat) or HFD (60% kcal as fat) with or without indicated dose of BL153 (2.5, 5, or 10 mg/kg body weight) for 24 weeks. (a) Body weight; (b) average energy intake for 12 weeks; (c) heart weight/tibia length ratio; and (d) insulin tolerance test (IPITT) as well as (e) area under the curves (AUC) after 20 weeks of control diet or HFD feeding. Data were presented as means ± SD (*n* = 5). ^a^ *P* < 0.05 versus control; ^b^ *P* < 0.05 versus HFD. Ctrl: control; HFD: high-fat diet.

HFD feeding gradually and significantly increased the body weight in HFD group compared to the control diet feeding group (Figure 1(a)). Low dose of BL153 (2.5 mg/kg) treatment had no significant effects on HFD feeding induced body weight increases compared to HFD group (Figure 1(a)). Both 5 mg/kg and 10 mg/kg of BL153 treatment slightly increased the body weight between 8 and 21 weeks compared to HFD group (Figure 1(a)). Energy intake for each group was calculated and it was found that the energy intake in HFD fed mice was significantly higher than that of control diet fed mice (Figure 1(b)). BL153 treatment had no significant effects on HFD-induced energy intake changes (Figure 1(b)). HFD feeding significantly increased heart weight (Figure 1(c)) and BL153 administration also had no significant effects on HFD increased heart weight (Figure 1(c)).

### 3.2. The Effects of BL153 on HFD-Induced Insulin Resistance

To evaluate the effects of long term BL153 administration on whole body glucose metabolism, IPITT was performed at 24 weeks after HFD feeding. HFD feeding significantly attenuated the insulin sensitivity in HFD group, while 5 mg/kg and 10 mg/kg of BL153 slightly prevented HFD-induced insulin sensitivity attenuation (Figures 1(d) and 1(e)).

### 3.3. The Effects of BL153 on HFD-Induced Cardiac Function Changes

Echo examination found that HFD feeding slightly, but not significantly, decreased cardiac function, including increases in LVID; d (LV end diastolic diameter), LVID; s (LV end systolic diameter), LV vol; s (LV end systolic volume), LV vol; d (LV end diastolic volume), and LV mass and decreases in ejection faction and fraction shortening in HFD group compared to control diet group. BL153 (10 mg/kg) administration prevented HFD-induced cardiac function changes (Table 1). Both HFD feeding and BL153 treatments had no significant effects on the diastolic, systolic, and mean BP and heart rate compared to control diet feeding group (Table 1).

### 3.4. BL153 Attenuated HFD-Induced Cardiac Lipid Accumulation

**Figure 2:**
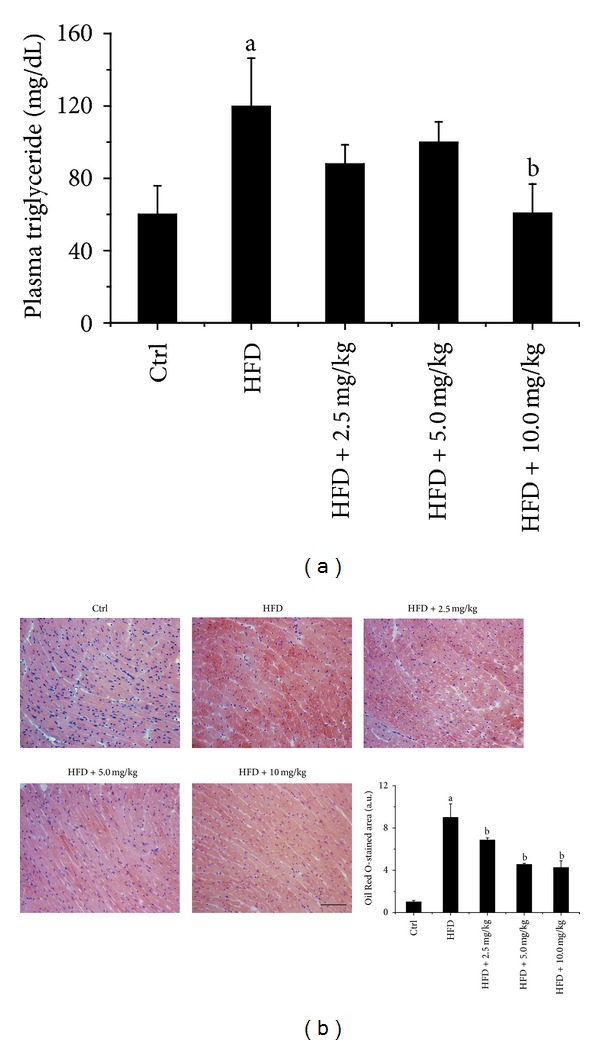
BL153 attenuated lipid accumulation induced by HFD in heart and plasma triglyceride. (a) Level of triglyceride in plasma was measured by a triglyceride colorimetric assay kit; (b) Lipid accumulation in heart was examined by Oil Red O staining (bar = 50 *μ*m). Data were presented as means ± SD (*n* = 5). ^a^ *P* < 0.05 versus control; ^b^ *P* < 0.05 versus HFD group.

Chronic obesity is associated with whole body and organ specific lipid metabolic disorder [23]. As expected, HFD feeding significantly increased triglyceride level in plasma in HFD group compared to the control diet feeding group. BL153 treatment significantly attenuated plasma triglyceride levels only at the dose of 10 mg/kg compared to HFD feeding group (Figure 2(a)). Correspondingly, Oil Red O staining revealed that HFD significantly increased cardiac lipid accumulation compared to control diet feeding group. BL153 administration at all dose levels significantly, but not completely, prevented HFD feeding-induced cardiac lipid accumulation compared to HFD fed group (Figure 2(b)).

### 3.5. BL153 Attenuated HFD-Induced Cardiac Inflammation

**Figure 3:**
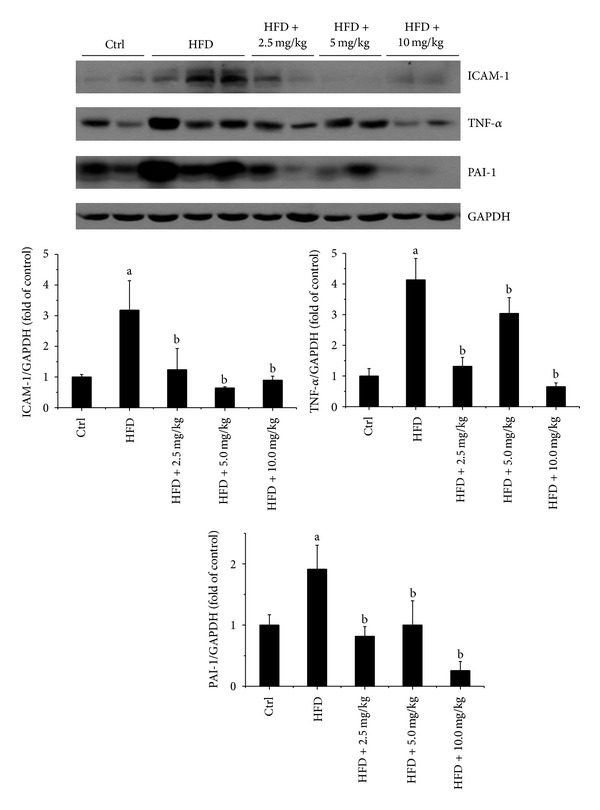
BL153 attenuated HFD-induced cardiac inflammation. The protein levels of inflammatory markers ICAM-1, TNF-*α*, and PAI-1 were examined by Western blot assay and quantified by densitometric analysis. GAPDH was used as loading control. Data were presented as means ± SD (*n* = 5). ^a^ *P* < 0.05 versus control group; ^b^ *P* < 0.05 versus HFD group.

Inflammation is elevated in obese rodents and humans, which is also an important contributor to the development of insulin resistance [24]. Thus, we detected the markers of inflammation in cardiac tissue. We found that HFD feeding significantly increased cardiac ICAM-1, TNF-*α*, and PAI-1 expression in HFD feeding mice (Figure 3), and BL153 significantly inhibited the inflammatory markers upregulation, while no significant differences between three dose levels of BL153 administration were observed (Figure 3).

### 3.6. BL153 Attenuated HFD-Induced Cardiac Oxidative Stress

**Figure 4:**
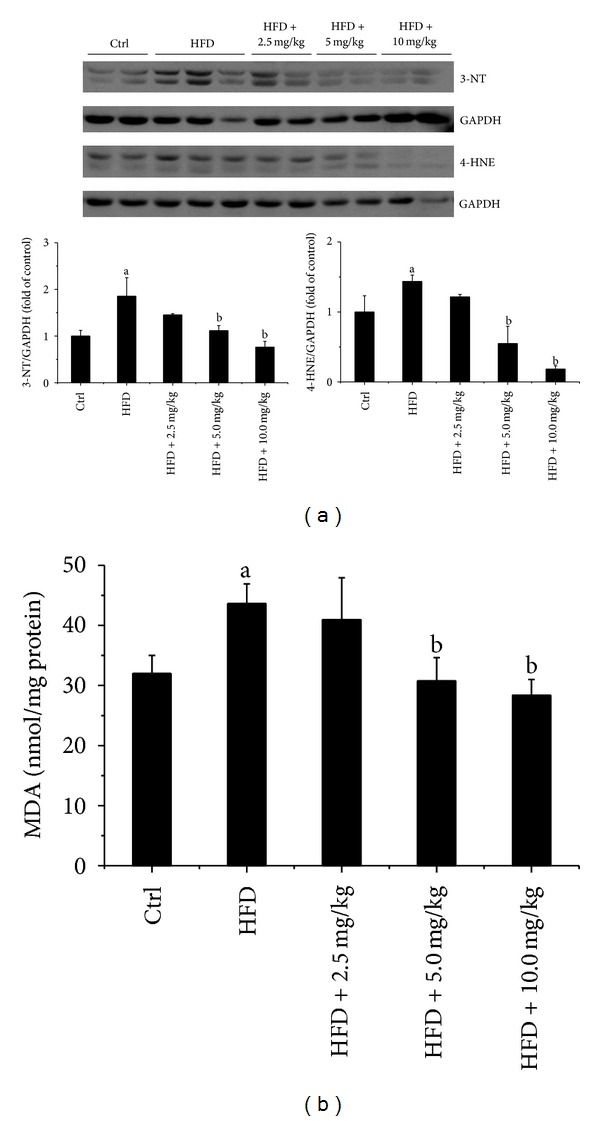
BL153 attenuated HFD-induced cardiac oxidative stress. (a) Cardiac oxidative stress was measured by Western blot examination of 3-NT and 4-HNE accumulation and quantified by densitometric analysis; (b) lipid peroxides (MDA) in cardiac tissue were evaluated by thiobarbituric acid reactivity assay. Data were presented as means ± SD (*n* = 5). ^a^ *P* < 0.05 versus control; ^b^ *P* < 0.05 versus HFD group.

Obesity is associated with oxidative stress in heart and liver [25]. So we investigated whether BL153 administration can prevent oxidative stress induced by HFD feeding. We measured the makers of oxidative stress including 3-NT, 4-HNE, and MDA, and found that the accumulation of 3-NT, 4-HNE (Figure 4(a)), and MDA (Figure 4(b)) in the heart was significantly enhanced by HFD feeding in HFD group, and both 5 mg/kg and 10 mg/kg of BL153 significantly prevented HFD-induced cardiac oxidative stress accumulation (Figures 4(a) and 4(b)).

### 3.7. BL153 Prevented HFD-Induced Cardiac Apoptotic Cell Death

**Figure 5:**
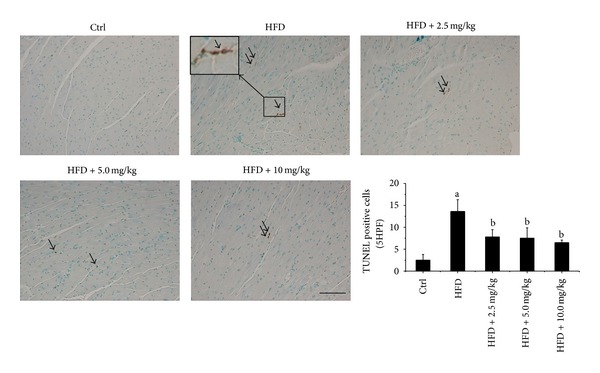
BL153 prevented HFD-induced cardiac apoptotic cell death. Myocardial apoptotic cell death was examined by TUNEL staining (Bar = 50 *μ*m). Arrows indicate TUNEL positive nuclei. The results were expressed as average number of TUNEL positive nuclei per five high power field under a microscopy. Data were means ± SD (*n* = 5). ^a^ *P* < 0.05 versus control; ^b^ *P* < 0.05 versus HFD group.

Reportedly, obesity related cardiac lipid accumulation and oxidative stress are important contributors to cardiac cell death and dysfunction in rodent and human [26]. In the current study, the cardiac cell death was detected by TUNEL staining (Figure 5). We found that HFD feeding significantly induced cardiac cell death in HFD fed mice, and BL153 treatment significantly, even not completely, prevented HFD-induced cardiac apoptotic cell death in all of the three dose levels of BL153 treated mouse.

**Figure 6:**
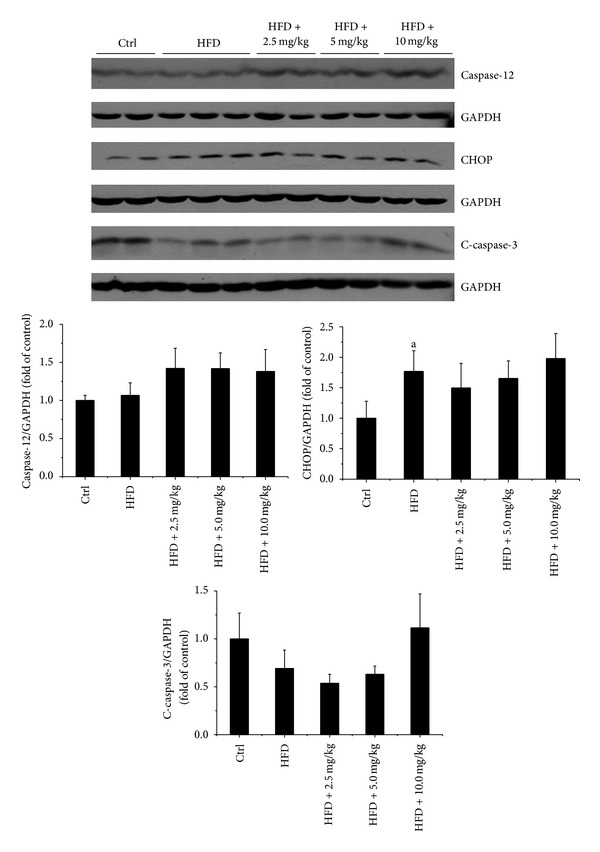
The effects of HFD and BL153 on ER stress-associated cell death makers. ER stress-associated cell death markers including CHOP, caspase-12, and C-caspase-3 (cleaved-caspase-3) were examined by Western blot assay and quantified by densitometric analysis. Data were presented as mean ± SD (*n* = 5). ^a^ *P* < 0.05 versus control; ^b^ *P* < 0.05 versus HFD group.

**Figure 7:**
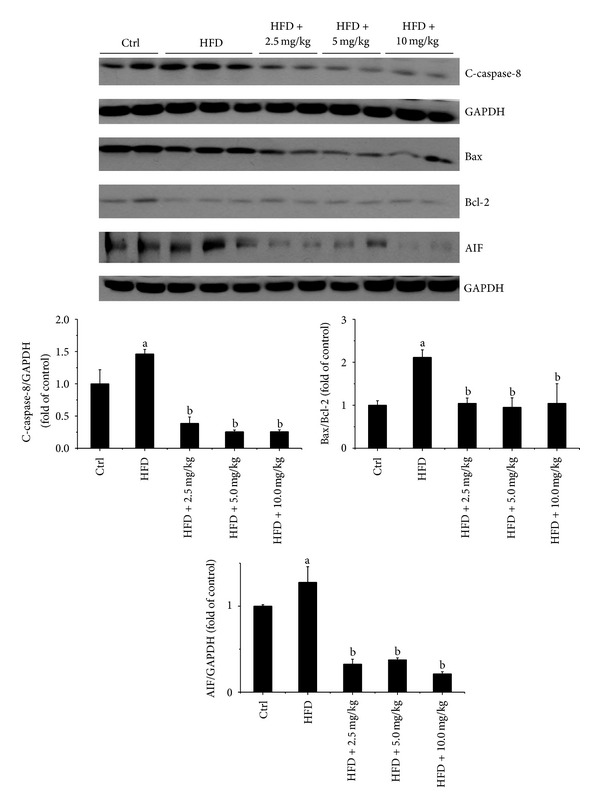
BL153 prevented HFD-induced cardiac apoptosis via activation of the mitochondrial cell death pathway. The expression of C-caspase-8 (cleaved-caspase-8), Bax, Bcl-2, and AIF in the heart was detected by Western blot assay and quantified by densitometric analysis. Data were presented as mean ± SD (*n* = 5). ^a^ *P* < 0.05 versus control. ^b^ *P* < 0.05 versus HFD group.

Endoplasmic reticulum (ER) and/or mitochondrial-associated cell death pathways may be involved in cardiac cell death under different pathological conditions [27, 28]. In order to investigate the signaling changes that may be involved in HFD-induced cardiac apoptotic cell death, both ER stress and mitochondrial-associated cell death pathway related molecular markers were examined by Western blot assay (Figures 6 and 7). Interestingly, HFD feeding both with and without BL153 treatment had no significant effects on the expression of ER stress related cell death markers including caspase-12, CHOP, and cleaved-caspase-3 (Figure 6), which indicated that HFD-induced cardiac apoptosis was independent of ER stress associated cell death signals. While HFD feeding significantly increased mitochondrial-associated cardiac cell death signals including the upregulation of cleaved-caspase-8 and Bax to Bcl-2 expression ratio in HFD group, and BL153 treatment completely prevented HFD-induced these cardiac cell death signals (Figure 7). The release of AIF from mitochondrial activated apoptotic cell death is independent of caspase-3 pathway [29]. The Western blot results revealed that HFD feeding significantly elevated cardiac AIF expression in HFD fed hearts and BL153 completely prevented HFD induced cardiac AIF upregulation (Figure 7), which indicates that HFD-induced cardiac cell death is predominantly attributed to mitochondrial-associated pathway and AIF/caspase-8/Bax/Bcl-2 apoptotic cascade is a critical target for BL153 prevention of HFD-induced cardiac cell apoptosis.

## 4. Discussion

The present study demonstrated that mice fed an HFD developed obesity phenotype, which was characterized by increased body weight, insulin resistance, and high level of triglyceride in the blood. The treatment of BL153 slightly ameliorated insulin resistance and decreased body weight. HFD feeding also resulted in significant lipid accumulation in the cardiac tissue accompanied by mild cardiac hypertrophy and slight cardiac function changes (Table 1), along with significant increases of cardiac inflammation (Figure 3), oxidative stress (Figure 4), and cardiac cell death (Figure 5), and the treatment of BL153 significantly prevented the above-mentioned cardiac detrimental changes (Figures 3–5).

Several studies have investigated cardiac lipotoxicity in HFD feeding induced and genetically manipulated rodent models [30–32]. Rats fed 60 kcal% fat diet for 6 weeks slightly elevated cardiac triglyceride levels [33]. We found a six fold increase in cardiac lipid accumulation by Oil Red O staining after 24 weeks of HFD feeding in mice, indicating that long term exposure to a HFD can induce significant cardiac lipid accumulation. Interestingly, treatment with BL153 significantly attenuated HFD feeding-induced cardiac lipid accumulation (Figure 2). This is the first study to show that BL153 exerts a function of cardiac lipid metabolic regulation in HFD-induced obesity.

Obesity is associated with cardiac hypertrophy and cardiac dysfunction, which is largely attributed to many pathophysiological conditions such as chronic inflammation, oxidative stress, mitochondrial dysfunction and apoptosis of cardiomyocytes [34–36]. HFD feeding-induced lipid accumulation evoked cardiac oxidative stress, inflammation and, eventually, cardiac dysfunction in animals [37]. It is known that the role of oxidative stress is postulated in many cardiovascular diseases [38]. Reactive oxygen species can promote inflammation, and *vice versa*, and both induce apoptotic cell death [39, 40]. *Magnolia* constituents including HON and MAG have been evaluated as antioxidants [41, 42] and anti-inflammation agents [10]. Our study also demonstrated for the first time that treatment with BL153 significantly ameliorated HFD-induced cardiac inflammatory responses including decreases of PAI-1, TNF-*α*, and ICAM-1 expression (Figure 3) and oxidative stress including decreases of 3-NT, 4-HNE, and MDA accumulation (Figure 4) and consequently prevented HFD-induced mild cardiac hypertrophy and cardiac function changes (Table 1). The anti-inflammatory and antioxidative mechanisms of *Magnolia *extract are proven to be associated with the decrease of nitric oxide (NO) production, the expression of inducible NO synthase (iNOS), TNF-*α*, and cyclooxygenases, and the activation of NF-*κ*B [43, 44].

A commonly proposed mechanism for lipid-induced cardiomyocyte death is via activation of caspases by ceramide, a potent proapoptotic by product of lipid metabolism, which elevates the expression of iNOS, thereby enhancing NO and peroxynitrite formation [45]. Incubation of cultured neonatal rat cardiomyocytes with palmitate causes a decrease in the ability of these cells to oxidize fatty acids, an increase in cellular malonyl-CoA, and a decrease in the activity of AMP-activated protein kinase (AMPK) compared to myocytes incubated in the presence of oleate [46]. While palmitate decreases the oxidative metabolism of fatty acids, it increases the formation of intracellular triglyceride and ceramide [46]. Increased ceramide formation is associated with an increase in apoptosis along with an increase in caspase-3 activity and DNA-laddering in many cell systems [46]. Our TUNEL staining results revealed that cardiac lipid accumulation significantly increased cardiac apoptotic cell death (Figure 5), which is consistent with the previous reports [47]. However, the protein levels of CHOP and caspase-12, as makers of ER stress, were similar in all groups (Figure 6). We also did not observe any significant change of caspase-3 cleavage (Figure 6). Therefore, the cardiac apoptotic cell death pathway induced by HFD in the present study may be ER stress and caspase-3 independent [48]. Relling et al. have demonstrated that HFD feeding-induced cardiac dysfunction was mitochondria dependent [48]. Our previous study also demonstrated that hyperglycemia-derived oxidative stress mediates a mitochondrial cytochrome c-mediated caspase-3 activation pathway playing an important role in diabetes-induced cardiac cell death [49, 50]. With the consistence, here cardiac lipid accumulation was found to induce cardiac apoptotic cell death associated with mitochondrial cell death pathway, shown by increased expression ratio of Bax to Bcl-2 expression along with the increased AIF expression. Several studies also have shown the fact that induction of cell death is caspase-3 independent in vitro and in vivo [51–53]. Taken together, the previous studies and our findings indicate that cardiac lipid accumulation induced cell death through upregulating inflammation and oxidative stress is associated with caspase-3 independent pathway.

Jin et al. demonstrated that MAG, one of the major bioactive constituents in *Magnolia* extract, at the dose of 10 mg/kg via intraperitoneal injection prevented myocardial ischemia and reperfusion injury through prevention of cardiomyocytes apoptosis [54]. Consistent with this study, our study also demonstrated for the first time that BL153 significantly inhibited HFD feeding-induced cardiac apoptotic cell death by prevention of cardiac lipid accumulation-induced cardiac inflammation and oxidative stress.

In summary, our study demonstrated that BL153 attenuates HFD-associated cardiac damage through prevention of HFD induced cardiac lipid accumulation, inflammation, oxidative stress, and mitochondria caspase-3 independent cell death pathway.

## Figures and Tables

**Table 1:** The effects of BL153 on cardiac function in HFD feeding mice.

	Control	HFD	HFD + 2.5 mg/kg	HFD + 5.0 mg/kg	HFD + 10 mg/kg
IVS; d (mm)	0.72 ± 0.03	0.71 ± 0.03	0.69 ± 0.02	0.70 ± 0.03	0.70 ± 0.0232
LVID; d (mm)	3.26 ± 0.18	3.61 ± 0.25	3.61 ± 0.17	3.68 ± 0.21	3.72 ± 0.23
LVPW; d (mm)	0.80 ± 0.10	0.85 ± 0.08	0.99 ± 0.08	0.89 ± 0.18	0.96 ± 0.10
IVS; s (mm)	1.10 ± 0.02	1.16 ± 0.15	1.03 ± 0.12	1.13 ± 0.17	1.19 ± 0.10
LVID; s (mm)	1.37 ± 0.23	1.72 ± 0.36	1.77 ± 0.35	1.82 ± 0.25	1.60 ± 0.19
LVPW; s (mm)	0.50 ± 0.24	1.48 ± 0.34	1.74 ± 0.25	1.55 ± 0.26	1.79 ± 0.20
LV vol; d (*μ*L)	43.16 ± 5.55	55.49 ± 9.08	55.05 ± 5.90	57.83 ± 8.07	59.40 ± 8.56
LV vol; s (*μ*L)	4.98 ± 2.04	9.40 ± 4.69	10.06 ± 4.73	10.54 ± 4.03	7.60 ± 2.29
%EF	88.76 ± 3.29	82.59 ± 10.14	81.71 ± 8.58	82.18 ± 4.45	87.44 ± 3.15
%FS	58.24 ± 4.59	52.25 ± 11.21	50.99 ± 10.22	50.80 ± 4.69	57.13 ± 4.15
LV mass (mg)	79.02 ± 3.31	97.10 ± 15.86	106.85 ± 13.03	101.68 ± 18.27	110.94 ± 18.34
HR (beats/min)	697.93 ± 53.40	677.98 ± 50.60	689.18 ± 34.90	675.86 ± 36.11	667.95 ± 27.90
Systolic BP (mmHg)	110.92 ± 5.13	113.09 ± 3.71	97.90 ± 11.34	98.79 ± 10.41	109.39 ± 5.48
Diastolic BP (mmHg)	79.69 ± 6.99	77.30 ± 5.65	69.75 ± 10.10	70.68 ± 10.27	79.34 ± 4.75
Mean BP (mmHg)	89.76 ± 6.35	88.92 ± 3.82	78.79 ± 10.46	79.73 ± 10.30	89.03 ± 4.84

*Notes*. IVS: interventricular septum; LVID; d: left ventricular end diastolic diameter; LVID; s: left ventricular end systolic diameter; LVPW: left ventricular posterior wall; EF: ejection fraction; FS: fractional shortening; LV vol; s: left ventricular end systolic volume; LV vol; d: left ventricular end diastolic volume; LV mass: left ventricular mass. HR: heart rate; BP: blood pressure. Data were presented as means ± SEM (*n* = 5).
